# Postprandial Glucose Level Decreases and Appetite in Adults Without Diabetes

**DOI:** 10.1001/jamanetworkopen.2026.3426

**Published:** 2026-03-26

**Authors:** Jiali Yao, Sarah M. Edney, Linda Wei Lin Tan, Shih Ling Kao, Xueling Sim, E Shyong Tai, Falk Müller-Riemenschneider, Rob M. van Dam

**Affiliations:** 1 Saw Swee Hock School of Public Health, National University of Singapore, Singapore, Singapore; 2 Yong Loo Lin School of Medicine, National University of Singapore, Singapore, Singapore; 3 University Medicine Cluster, National University Health System, Singapore, Singapore; 4 Berlin Institute of Health, Charité-Universitätsmedizin Berlin, Berlin, Germany; 5 Milken Institute School of Public Health, The George Washington University, Washington, DC; 6 Harvard T. H. Chan School of Public Health, Boston, Massachusetts

## Abstract

**Importance:**

Biomarkers that can predict appetite and be monitored in daily life could guide appetite and weight management. Glucose level declines induce hunger in laboratory settings, but free-living data are limited.

**Objective:**

To characterize postprandial glucose level decreases (PGDs) 2 to 3 hours following free-choice meals and estimate the association of PGDs with appetite.

**Design, Setting, and Participants:**

This cohort study, conducted from May 2021 to August 2024, used intensive longitudinal data from smartphone app–based ecologic momentary assessments (EMA) and continuous glucose monitoring (CGM) over 9 days among Singaporean adults without diabetes. Data were analyzed between November 2024 and August 2025.

**Exposures:**

Two CGM-derived PGD measures per meal: PGD magnitude (percentage of glucose level reduction 2 to 3 hours after meals compared with the premeal baseline level) and PGD below the baseline level (binary indicator coded as yes when PGD magnitude was >0).

**Main Outcomes and Measures:**

The main outcomes were 6 EMA-derived appetite measures, including hunger level and hunger increase 2 to 3 hours and 3 to 4 hours after meals, time to the next meal, and time to the next meal or snack. Multivariable generalized estimating equation models estimated the adjusted association for each pair of PGD exposure (PGD magnitude, PGD below the baseline level) and the appetite measure.

**Results:**

The analysis included 7650 meals from 895 participants (mean [SD] age, 40.1 [13.6] years; 561 [63%] female). Median time to the next meal was 380 (IQR, 285-660) minutes when PGD was below the baseline level (4121 meals [54%]) compared with 425 (IQR, 325-714) minutes when PGD below the baseline level was absent. Larger PGD magnitudes were associated with greater postprandial hunger levels at 2 to 3 hours (β = 0.05 [95% CI, 0.03-0.07] per 10% decrease) and 3 to 4 hours (β = 0.09 [95% CI, 0.06-0.13] per 10% decrease), greater hunger increases at 2 to 3 hours (β = 0.08 [95% CI, 0.04-0.12] per 10% decrease) and 3 to 4 hours (β = 0.07 [95% CI, 0.02-0.12] per 10% decrease), and shorter time to the next meal (β = −6.30 [95% CI, −8.88 to −3.72] minutes) and the next meal or snack (β = −6.54 [95% CI, −8.88 to −3.72] minutes). Similarly, PGD below the baseline level was associated with all appetite measures, including a shorter time to the next meal (β = −27.30 [95% CI, −36.90 to −17.71] minutes).

**Conclusions and Relevance:**

In this cohort study of adults without diabetes, PGD was associated with greater perceived hunger and earlier subsequent eating. Our findings support PGD as a potential biomarker for appetite regulation and target for weight management.

## Key Points

**Question:**

Are postprandial glucose level decreases associated with appetite among free-living adults without diabetes?

**Findings:**

In this cohort study of 7650 meals from 895 participants, postprandial glucose level decreases in the 2- to 3-hour period after meals varied widely. Postprandial glucose level decreases were associated with greater postprandial hunger at 2 to 3 hours and 3 to 4 hours and with a shorter time to the initiation of the next food intake.

**Meaning:**

Our findings support postprandial glucose level decrease as a potential biomarker to enhance understanding of appetite regulation and as a potential target for appetite and weight management.

## Teaser

This cohort study examines the association between postprandial glucose level decreases and appetite regulation among adults without diabetes in Singapore.

## Introduction

Obesity is associated with substantial increases in the risk of type 2 diabetes, cardiovascular diseases, several types of cancer, and arthritis, contributing to disability and premature mortality.^1^ The rising prevalence of obesity, affecting more than 1 billion people worldwide, underscores an urgent need for effective weight control.^1,2^ Appetite suppression is a key mechanism for weight management, but developing sustainable nonpharmacologic strategies to modulate appetite continues to pose a major challenge.^1,3,4^

Glucose is the human body’s primary energy source. Hypoglycemia triggers counterregulatory responses, including increased hunger and food-seeking behaviors.^5,6,7^ Modest declines in blood glucose level have also been shown to stimulate appetite in controlled laboratory experiments.^6,8,9^ However, these studies may lack external validity, and free-living studies on this topic remain scarce.^9^ The study by Wyatt et al^10^ was an exception, investigating postprandial glucose levels and appetite in more than 1000 UK and US adults without diabetes under free-living conditions. The authors found that postprandial glucose level decreases (PGDs) in the 2 to 3 hours after standardized breakfast meals were associated with increased hunger and subsequent energy intake.^10^ While standardized meals strengthen internal validity and mechanistic clarity, they compromise ecologic validity, which can be addressed by free-choice meals. It also remains unclear whether findings from breakfast generalize to other meals. To our knowledge, the relationship between PGD and appetite has not been examined for free-choice meals consumed throughout the day. In addition, characteristics of PGDs remain largely unexplored.

In this cohort study, we used a combination of masked continuous glucose monitoring (CGM) and smartphone app–based ecologic momentary assessment (EMA) surveys to capture intensive, real-time, free-living data on glycemic dynamics, appetite, and diet behaviors over a 9-day period in a multiethnic cohort of Singaporean adults without diabetes. We aimed to examine the characteristics of PGDs following free-choice meals throughout the day and to estimate the association of PGD with postprandial hunger and time to initiating subsequent eating.

## Methods

### Study Population

Participants were from the Continuous Observations of Behavioral Risk Factors in Asia (COBRA) study, a longitudinal cohort study nested within the Singapore Multi-Ethnic Cohort (MEC). MEC is a population-based cohort of community-dwelling adults from the 3 major ethnic groups (Chinese, Indian, and Malay) in Singapore; the MEC study has been conducted since 2004.^11,12^ Recruitment was through public outreach and referrals, with ongoing enrollment to enhance representativeness of the general Singapore population except for a targeted oversampling for Indian and Malay individuals (the ethnic minority groups).^11,12^ Between May 2021 and August 2024, eligible MEC participants were invited to participate in COBRA. The COBRA study protocol, guided by the Strengthening the Reporting of Observational Studies in Epidemiology (STROBE) checklist and the Checklist for Reporting EMA Studies, has been published elsewhere.^13,14^ In brief, participants were male and female Singapore residents aged 21 to 69 years of Chinese, Indian, or Malay ethnicity (as recorded on their Singapore National Registration Identity Card). Individuals with relevant medical constraints or major chronic diseases were excluded (eMethods in Supplement 1). All participants provided written informed consent. Ethical approval was obtained from the institutional review board of the National University of Singapore. This study followed the STROBE reporting guideline.

### Study Design

Participants underwent a baseline visit, including a questionnaire interview and physical examination, at the National University of Singapore (eFigure 1 in Supplement 1). Participants’ sociodemographic and medical information was collected at the baseline visit. This visit was followed by 9 free-living days of continuous monitoring using mobile technologies, including smartphone app–based EMA surveys and masked CGM devices. During the 9 days, participants were asked to maintain their usual daily behaviors.

An app (Avicenna; Avicenna Research Inc) was installed on participants’ smartphones at the baseline visit. During the free-living days, the app delivered 6 EMA surveys per day to each participant at random times within 6 time intervals across the day (8:00-9:30 am, 10:30 am to 12:00 pm, 1:00-2:30 pm, 3:30-5:00 pm, 6:00-7:30 pm, and 8:30-9:30 pm). Participants were instructed to record all food intake in the EMAs throughout the 9 days and report the time (in hour: minute format) for each meal and snack. The first EMA of the day prompted participants to report their food intake for 2 specific periods: the period between the last EMA and bedtime the previous day (applicable only on the second to ninth days) and the period since waking. The subsequent 5 EMAs of the day asked about food intake since the previous EMA. Each EMA also collected participants’ momentary hunger levels, with the response time automatically recorded by the app.

At the baseline visit, each participant was fitted with a CGM device (Freestyle Libre Pro iQ; Abbott Diabetes Care) on their nondominant arm. Throughout the free-living days, the CGM automatically measured the interstitial glucose level every 15 minutes and was masked from participants.

### Quality Control for Meal Inclusion

Meals were included in the primary analysis if all the following criteria were met: (1) meal was consumed by the time of the sixth EMA of the day, (2) meal was consumed after 5:00 am, (3) participant had sufficient EMA data to assess their status (eg, fasting and appetite status) within the period around the meal (±4 hours), (4) participant had at least 2 hours of premeal fasting and at least 3 hours of postmeal fasting, and (5) participant had complete time-matched CGM data during the 30 minutes before and 3 hours after the meal. Details are given in eFigure 2 in Supplement 1.

### Appetite Measures

In each EMA, participants responded to the question, “How hungry do you feel right now?” by a visual analog scale from 0 (not at all) to 6 (very much) adapted from Flint et al^15^ and Cardello et al.^16^ This question was used to extract hunger levels before and after each meal. We defined premeal hunger level as the most recent reported hunger within 2 hours before the meal. We defined postprandial hunger levels at 2 to 3 hours as the earliest reported hunger during the 2 to 3 hours after the meal. Subsequently, we calculated postprandial hunger increases at 2 to 3 hours by subtracting the premeal hunger level from postprandial hunger levels. Postprandial hunger levels and hunger increases at 3 to 4 hours were computed similarly but were restricted to meals with at least 4 hours of postmeal fasting. For each meal, we also calculated the time to the next meal and the time to the next meal or snack using the intervals between the current mealtime and the subsequent food intake within a 16-hour period. Data from all meal and snack records, including meals excluded from the main analysis, were used to compute the time intervals.

### Glucose Measures

Meal-specific glycemic measures were computed using CGM data. We calculated the premeal baseline glucose level specific to each meal using the mean glucose level 30 minutes before the meal (sensitivity analysis in eMethods in Supplement 1). Subsequently, we calculated 2 PGD measures using the baseline glucose level and glucose levels collected 2 to 3 hours after the meal. PGD magnitude was the percentage of glucose level reduction at 2 to 3 hours compared with the premeal baseline level: ([baseline glucose level – the lowest glucose level at 2-3 hours] / baseline glucose level) × 100%. PGD below the baseline level was a binary variable indicating whether postprandial glucose levels at 2 to 3 hours were below the baseline level (yes if the PGD magnitude was >0 and no if not). In addition, we used the baseline glucose level and glucose levels at 0 to 2 hours to calculate the postprandial glucose incremental area under the curve (iAUC) at 0 to 2 hours using the trapezoidal rule and glucose level increase magnitude: ([peak glucose level at 0-2 hours – baseline glucose level] / baseline glucose level) × 100%.^17^

### Statistical Analysis

We computed Spearman correlation coefficients between postprandial glucose measures and appetite measures (meals as observations). Because of the clustered nature of the meal data, the 95% CI for each correlation coefficient was computed using 10 000 bootstrapping samples of the original meal data to assess statistical significance (eMethods in Supplement 1).

We performed multivariable generalized estimating equation (GEE) models to estimate the adjusted association for each pair of PGD exposure (ie, PGD magnitude and PGD below the baseline level) and the appetite measure (dependent variable) because these models can account for within-participant correlations in the repeated-meal data and provide robust estimates. All models used meals as observations and participants as clusters using the R package geepack, version 1.3.12 (R Project for Statistical Computing) with a Gaussian identity link function. Statistical inference was based on robust standard errors (sandwich estimates). Covariates in the main models were mealtime (5:00-11:00 am, 11:00 am to 5:00 pm, and 5:00 pm to 12:00 am) and participant characteristics, including age (years), sex (male and female), ethnicity (Chinese, Indian, and Malay), glycemic status (normoglycemia and prediabetes), body mass index (BMI; calculated weight in kilograms divided by height in meters squared), cigarette smoking (yes or no), heavy alcohol consumption (yes or no), and the highest educational attainment (below A-level, A-level or equivalent, and university or above). Sensitivity models were performed with further adjustment for postmeal satiety, postprandial glucose iAUC, and temporal lifestyle factors, including meal composition, physical activity, and sleep (eMethods in Supplement 1). Furthermore, we applied the person-mean centering approach to extract the within-person variability measure for each PGD measure. The within-person variability measure was then used to replace the original PGD measure in the main models to estimate associations between within-person changes in PGD and appetite. These within-person analyses were restricted to individuals with at least 2 qualified meals with the respective appetite data. Additionally, we conducted sensitivity analyses using log-transformed Gaussian GEE models and untransformed Gamma (log link) GEE models for time-based appetite measures. We also performed models with additional interaction terms of PGD with mealtime, participant glycemic status, and weight status (BMI <23, ≥23 and <27.5, and ≥27.5). The respective stratified models were also performed.

All analyses were conducted in R, version 4.4.2 between November 2024 and August 2025. Hypothesis tests were 2-sided, and *P* values less than 0.05 were considered statistically significant.

## Results

Our study included 895 participants (561 [63%] female and 334 [37%] male) with a mean (SD) age of 40.1 (13.6) years (Table). The ethnic composition of participants was 583 (65%) Chinese, 130 (15%) Indian, and 182 (20%) Malay. Furthermore, 661 participants (74%) had normoglycemia, and 234 (25%) had prediabetes. The participants responded to 44 877 scheduled EMA surveys (93%) and recorded 15 128 meals. In total, 7650 meals met the inclusion criteria for the current analysis. These meals were nearly evenly distributed across breakfast time (2163 [28%] at 5:00-11:00 am), lunch time (2850 [37%] at 11:00 am to 5:00 pm), and dinner time (2637 [34%] at 5:00 pm to 12:00 am). Meal details are summarized in eTable 1 in Supplement 1.

**Table.** Participant Characteristics

Characteristic	Participants (N = 895)^a^
Age, mean (SD), y	40.1 (13.6)
Sex	
Female	561 (63)
Male	334 (37)
Ethnicity	
Chinese	583 (65)
Indian	130 (15)
Malay	182 (20)
Glycemic status	
Normoglycemia	661 (74)
Prediabetes^b^	234 (26)
Current cigarette smoker	114 (13)
Heavy alcohol consumer^c^	74 (8)
Current worker	711 (79)
BMI, mean (SD)	24.7 (5.5)
Fasting plasma glucose level, mean (SD), mg/dL	90.4 (8.2)
HbA_1c_, mean (SD), %	5.4 (0.3)

Abbreviations: BMI, body mass index (calculated weight in kilograms divided by height in meters squared); HbA_1c_, glycated hemoglobin.

SI conversion factor: To convert plasma glucose to millimoles per liter, multiply by 0.0555.

^a^ Data are presented as number (percentage) of participants unless otherwise indicated.

^b^ Defined as no diabetes but an HbA_1c_ level of 5.7% or greater or a fasting plasma glucose level of 100.0 mg/dL or greater.

^c^ Male participants having at least 5 servings of alcohol or female participants having at least 4 servings in a single drinking session.

### Patterns of PGDs

**Figure 1.**
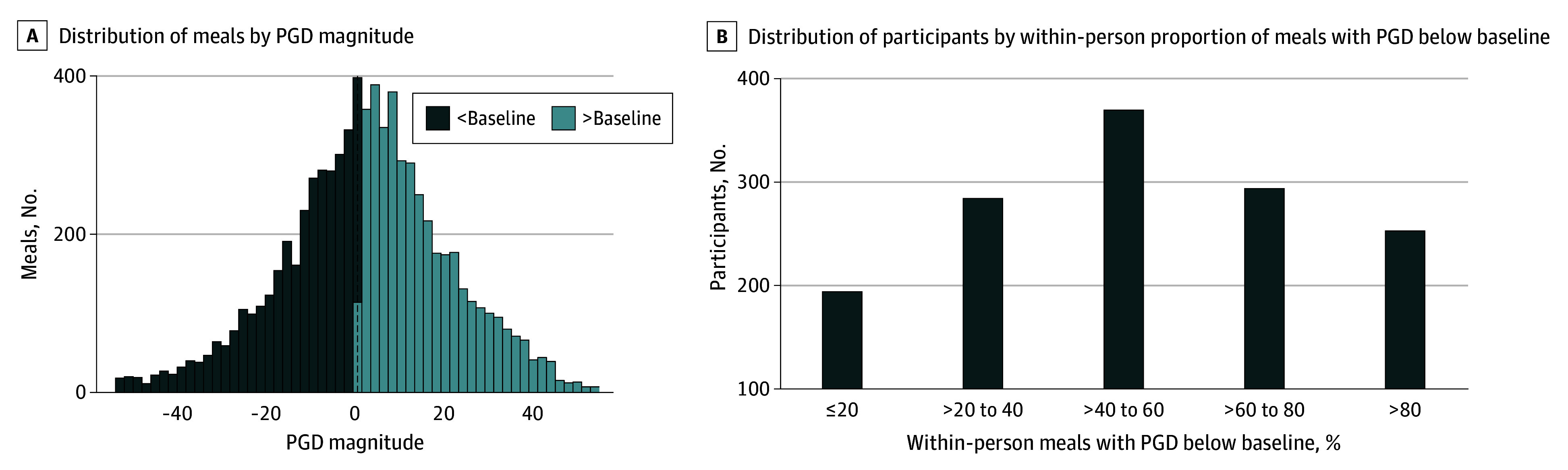
Histogram and Bar Graph of Postprandial Glucose Level Decreases (PGDs) in 7650 Meals From 895 Participants A, PGD magnitude was the percentage of glucose level reduction 2 to 3 hours after meals compared with the premeal baseline level. B, PGD below baseline was a binary indicator coded as yes when PGD magnitude was greater than 0.

As shown in Figure 1A, PGD magnitude (2-3 hours) varied widely across meals, with a mean (SD) of 0.7% (22.1%). Although glucose levels in 7320 meals (96%) did not fall below 70.0 mg/dL (to convert to millimoles per liter, multiply by 0.0555), PGD below the baseline level was observed in 4121 meals (54%), with a range from 1404 (49%) to 1324 (61%) across subgroups stratified by mealtime, participant glycemic status, and weight status (eTable 2 in Supplement 1). The within-person proportion of meals with PGD below the baseline level also varied widely among individuals (Figure 1B and eFigure 3 in Supplement 1). Among the participants, 94 (11%) experienced PGD below the baseline level in 20% or fewer meals, and 153 (17%) experienced it in over 80% of meals.

### PGD and Appetite

**Figure 2.**
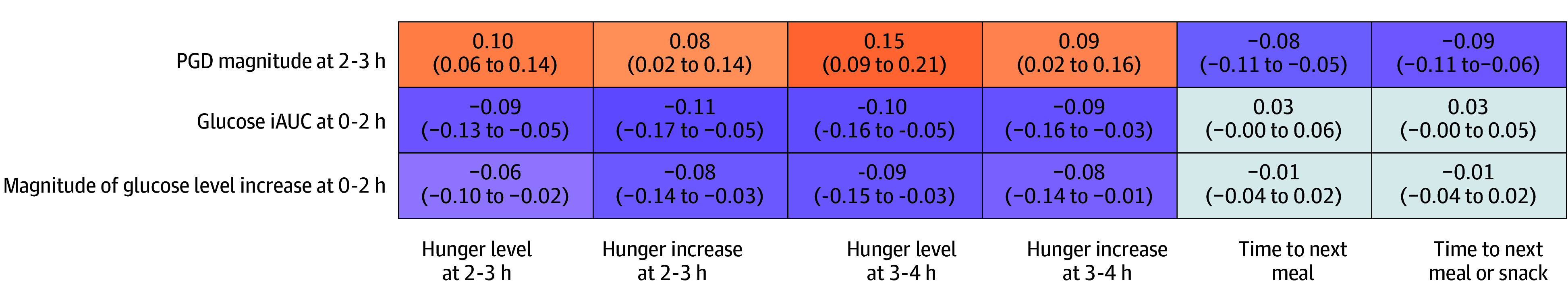
Spearman Correlations Between Glucose Level Measures and Appetite Measures With Meals as Observations Due to the clustered nature of the meal data, 95% CIs were estimated from the correlation distributions based on 10 000 bootstrapping samples of the original meal data (each bootstrapping sample containing meals from 895 participants). Orange indicates a positive correlation between the glucose level measure and the appetite measure and purple, a negative correlation. iAUC indicates incremental area under the curve; PGD, postprandial glucose level decrease.

Figure 2 shows the unadjusted correlation coefficients for PGD magnitude, glucose iAUC (0-2 hours), and magnitude of glucose level increase (0-2 hours) and the 6 appetite measures. PGD magnitude was consistently correlated with all the appetite measures. In addition, PGD magnitude was inversely correlated with glucose iAUC (Spearman ρ = −0.62; 95% CI, −0.64 to −0.60) and magnitude of glucose level increase (Spearman ρ = −0.59; 95% CI, −0.61 to −0.57). However, PGD magnitude had stronger correlations with all appetite measures except hunger increase at 2 to 3 hours compared with glucose iAUC and magnitude of glucose level increase. Appetite measures stratified by PGDs below the baseline level are summarized in eTable 3 in Supplement 1.

**Figure 3.**
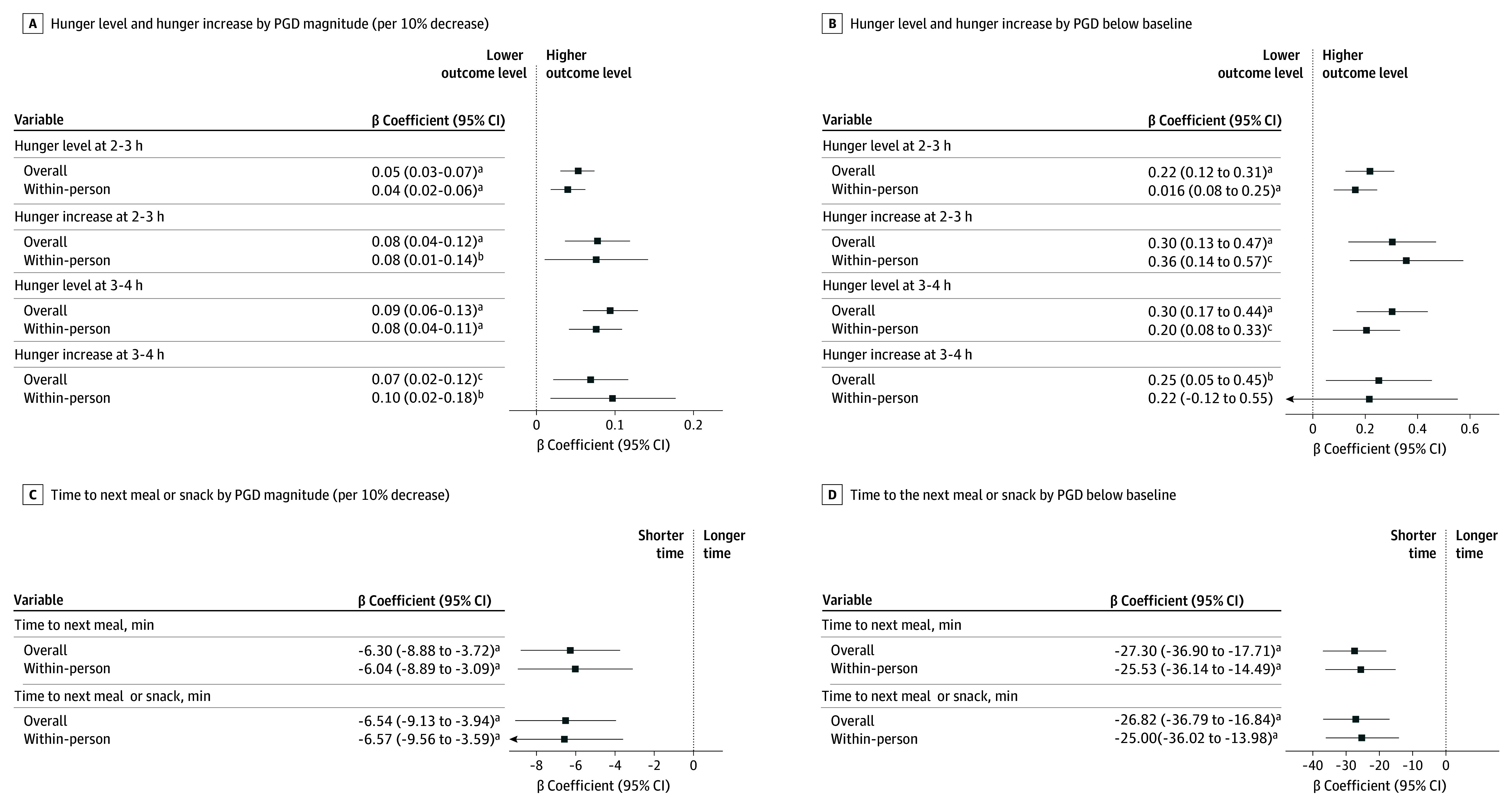
Dot-and-Whisker Plots of Overall and Within-Person Associations of Postprandial Glucose Level Decreases (PGDs) at 2 to 3 Hours With Appetite Measures The associations were estimated using generalized estimating equation models adjusted for age, sex, ethnicity, glycemic status, body mass index, cigarette smoking, alcohol consumption, educational level, and mealtime. ^a^*P* < .001. ^b^*P* < .05. ^c^*P* < .01.

Figure 3 and eTable 4 in Supplement 1 show the adjusted associations between PGDs and appetite measures. A higher PGD magnitude was associated with greater postprandial hunger levels at 2 to 3 hours (β = 0.05 [95% CI, 0.03-0.07] per 10% decrease) and 3 to 4 hours (β = 0.09 [95% CI, 0.06-0.13] per 10% decrease), greater postprandial hunger increases at 2 to 3 hours (β = 0.08 [95% CI, 0.04-0.12] per 10% decrease) and 3 to 4 hours (β = 0.07 [95% CI, 0.02-0.12] per 10% decrease), and shorter time to the next meal (β = −6.30 [95% CI, −8.88 to −3.72] minutes) and the next meal or snack (β = −6.54 [95% CI, −8.88 to −3.72] minutes). Similarly, PGD below the baseline level was associated with all of these appetite measures, including a shorter time to the next meal (β = −27.30 [95% CI, −36.90 to −17.71] minutes). These findings remained robust in sensitivity analyses, including models assessing within-person associations using individuals as their own control, models with further adjustment for postmeal satiety, postprandial glucose iAUC, or temporal lifestyle factors and models applying log transformation or Gamma distribution (Figure 3 and eTables 4-6 in Supplement 1).

Results of interaction and subgroup analyses are shown in eTable 7 and eFigures 4 to 6 in Supplement 1. The association between PGD magnitude and postprandial hunger increase at 2 to 3 hours was modified by mealtime (*P* = .04 for interaction for lunch vs breakfast; *P* = .003 for interaction for dinner vs breakfast). PGD magnitude was significantly associated with greater hunger increases 2 to 3 hours after lunch and dinner but not after breakfast. No modification of associations was observed by participant glycemic or weight status.

## Discussion

In this cohort study using high-resolution, free-living measurements, we characterized glucose level decreases 2 to 3 hours after free-choice meals throughout the day and examined the association of these decreases with postprandial appetite. We found substantial variability in PGDs across the 7650 meals and 895 individuals, with PGDs below the premeal baseline level observed for 54% of the meals. Greater PGD was associated with greater hunger levels and increases 2 to 3 hours and 3 to 4 hours after meals as well as shorter time to the subsequent eating episode.

Human appetite is influenced by a wide range of internal physiologic and psychologic factors as well as external environmental factors.^9^ Among these, the role of glucose in appetite regulation was formalized in the glucostatic theory in 1953 and has since been extensively studied in animals and humans.^6,9,18^ These studies found that relative declines in blood glucose level rather than absolute concentrations (except in cases of hypoglycemia) are closely linked to hunger and precede meal initiation.^5,6,7,9^ Due to practical constraints with small sample sizes, most past studies were conducted in highly controlled conditions. These studies provided valuable insights into internal appetite-driving signals but were limited in external validity. Addressing the scarcity of knowledge, Wyatt et al^10^ reported that glucose level decreases occurring 2 to 3 hours after the standardized breakfast meals were associated with greater hunger increases at 2 to 3 hours and with energy intake at 3 to 4 hours and throughout the day among 1070 free-living UK and US adults. Consistent with the laboratory evidence and the findings of Wyatt et al,^10^ our study showed that both higher PGD magnitudes and PGD below the baseline level were associated with greater postprandial hunger and faster initiation of subsequent food intake in a multiethnic Asian population. Our findings advance the evidence base by addressing the important gaps in observational investigations on free-choice meals and nonbreakfast meals. Collectively, the evidence suggests that glucose level decreases are associated with appetite in the general adult population even amid pervasive environmental cues that affect appetite.^9,10^ Of note, the interaction between mealtime and PGD magnitude was significant for hunger increase at 2 to 3 hours. Given that this result was not observed for PGD below the baseline level or for the other appetite measures, it may reflect a chance finding and should be confirmed in future studies.

Furthermore, PGD magnitude had a stronger correlation with appetite measures than with conventional postprandial hyperglycemia measures, such as glucose iAUC and glucose level increase at 0 to 2 hours.^10^ The growing accessibility of CGM also enables more feasible tracking of glucose level decreases in daily life compared with other physiologic appetite markers, such as glucagon-like peptide 1, glucose-dependent insulinotropic polypeptide, and ghrelin.^9^ These findings together suggest that PGD may serve as a practical biomarker to improve understanding of the regulation of appetite by blood glucose in daily life.

Further research is needed to clarify the underlying mechanisms and determine whether PGD can serve as a target for appetite and weight management interventions. However, our findings may be particularly relevant, as CGMs have become increasingly available and affordable, potentially allowing individuals to use CGMs for a period to identify their triggers for glucose level decreases and inform strategies for appetite and weight control.^19^

### Strengths and Limitations

In addition to investigating free-choice meals throughout the day in the general population on a large scale, our study has several other strengths. First, our study leveraged mobile technologies to collect real-time data on glycemic dynamics, appetite, and dietary factors. Unlike traditional approaches relying heavily on recalls, we used smartphone app–based EMA surveys to frequently capture appetite and food consumption throughout the day and CGM to record objective glycemic levels. These intensive longitudinal data also enabled analysis at meal-level resolution and within-person analyses that minimized confounding by between-person differences. Second, our study achieved good participant adherence with the free-living measurements, with a response rate of 93% for the EMA surveys. Third, prior research in this field has largely been conducted in Western populations, and our study provides insights into Asian populations.

Our study also has several limitations. First, our appetite measures were derived from self-reported hunger and timing of food consumption rather than objective measurements. Although masked CGM prevented biases due to awareness of glucose values, nondifferential misclassification and measurement errors may have persisted, leading to underestimated effect sizes. More precise assessments may detect larger effect sizes. Second, we derived the premeal hunger level using the most recent hunger level reported within 2 hours before the meal. This wide assessment time window may compromise the precision of the derived hunger increase (2 of 6 appetite measures). However, a sensitivity analysis restricting the measure of premeal hunger to within 1 hour before the meal yielded consistent findings (eTable 4 in Supplement 1). Third, our study could not account for other well-established appetite biomarkers, such as glucagon-like peptide-1 and glucose-dependent insulinotropic polypeptide, due to technical challenges in monitoring them in the general population in daily life.^9^ Future studies that overcome the methodologic barriers could yield deeper insights. Considering the limitations, the findings of the present study should be interpreted with caution pending further confirmation.

## Conclusions

This cohort study found that PGDs were associated with increased appetite after free-choice meals among adults without diabetes. PGD reductions were also associated with reduced hunger after meals and later subsequent eating episodes. Our findings support the use of PGD as a potential biomarker to enhance the understanding of appetite regulation.

## Notes

Supplement 1.
eMethods.
**eTable 1.** Meal Characteristics, Overall, and by Mealtime **eTable 2.** Postprandial Glucose Level Decreases (PGD) Stratified by Participant Glycemic Status, Weight Status, and Mealtime **eTable 3.** Meal Characteristics: Appetite Measures by Postprandial Glucose Dip Below Baseline **eTable 4.** Associations of Postprandial Glucose Level Decreases With Appetite Measures, Main, and Sensitivity Analyses **eTable 5.** Associations of Postprandial Glucose Level Decreases With Time to the Next Meal: Main Model vs Sensitivity Models With Log-Transformation and Models With Gamma Distribution **eTable 6.** Associations of Postprandial Glucose Level Decreases With Time to the Next Meal or Snacks: Main Model vs Sensitivity Models With Log-Transformation And Models With Gamma Distribution **eTable 7.** Interactions by Mealtime, Glycemic Status, and Weight Status for Associations Between Postprandial Glucose Level Decreases and Appetite **eFigure 1.** Study Design **eFigure 2.** Meal and Participant Inclusion and Flowchart **eFigure 3.** Bar Plot of Participants’ Proportion of Meals With Postprandial Glucose Level Decreases (PGD) Below Baseline **eFigure 4.** Stratified Associations of Postprandial Glucose Level Decreases (PGD) at 2 to 3 Hours With Appetite Measures by Mealtime **eFigure 5.** Stratified Associations of Postprandial Glucose Level Decreases (PGD) at 2 to 3 Hours With Appetite Measures by Glycemic Status **eFigure 6.** Stratified Associations of Postprandial Glucose Level Decreases (PGD) at 2 to 3 Hours With Appetite Measures by Body Mass Index Groups

Supplement 2.
Data Sharing Statement
