# Supplementary material for: Postprandial Glucose Level Decreases and Appetite in Adults Without Diabetes
Source: JAMA Netw Open. 2026 Mar 26;9(3):e263426. doi: 10.1001/jamanetworkopen.2026.3426 (PMC13022734; doi:10.1001/jamanetworkopen.2026.3426)
Supplement: Supplement 1. — eMethods. eTable 1. Meal Characteristics, Overall, and by Mealtime eTable 2. Postprandial Glucose Level Decreases (PGD) Stratified by Participant Glycemic Status, Weight Status, and Mealtime eTable 3. Meal Characteristics: Appetite Measures by Postprandial Glucose Dip Below Baseline eTable 4. Associations of Postprandial Glucose Level Decreases With Appetite Measures, Main, and Sensitivity Analyses eTable 5. Associations of Postprandial Glucose Level Decreases With Time to the Next Meal: Main Model vs Sensitivity Models With Log-Transformation and Models With Gamma Distribution eTable 6. Associations of Postprandial Glucose Level Decreases With Time to the Next Meal or Snacks: Main Model vs Sensitivity Models With Log-Transformation And Models With Gamma Distribution eTable 7. Interactions by Mealtime, Glycemic Status, and Weight Status for Associations Between Postprandial Glucose Level Decreases and Appetite eFigure 1. Study Design eFigure 2. Meal and Participant Inclusion and Flowchart eFigure 3. Bar Plot of Participants’ Proportion of Meals With Postprandial Glucose Level Decreases (PGD) Below Baseline eFigure 4. Stratified Associations of Postprandial Glucose Level Decreases (PGD) at 2 to 3 Hours With Appetite Measures by Mealtime eFigure 5. Stratified Associations of Postprandial Glucose Level Decreases (PGD) at 2 to 3 Hours With Appetite Measures by Glycemic Status eFigure 6. Stratified Associations of Postprandial Glucose Level Decreases (PGD) at 2 to 3 Hours With Appetite Measures by Body Mass Index Groups [file jamanetwopen-e263426-s001.pdf]

Supplementary Online Content

Yao J, Edney SM, Tan LWL, et al. Postprandial glucose level decreases and appetite in adults without diabetes. *JAMA Netw Open*. 2026;9(3):e263426.
doi:10.1001/jamanetworkopen.2026.3426

eMethods
eTable 1. Meal Characteristics, Overall, and by Mealtime
eTable 2. Postprandial Glucose Level Decreases Stratified by Participant Glycemic Status, Weight Status, and Mealtime
eTable 3. Meal Characteristics: Appetite Measures by Postprandial Glucose Level Decreases Below Baseline
eTable 4. Associations of Postprandial Glucose Level Decreases With Appetite Measures, Main, and Sensitivity Analyses
eTable 5. Associations of Postprandial Glucose Level Decreases With Time to the Next Meal: Main Model vs Sensitivity Models With Log-Transformation and Models With Gamma Distribution
eTable 6. Associations of Postprandial Glucose Level Decreases With Time to the Next Meal or Snacks: Main Model vs Sensitivity Models With Log-Transformation And Models With Gamma Distribution
eTable 7. Interactions by Mealtime, Glycemic Status, and Weight Status for Associations Between Postprandial Glucose Level Decreases and Appetite
eFigure 1. Study Design
eFigure 2. Meal and Participant Inclusion and Flowchart
eFigure 3. Bar Plot of Participants' Proportion of Meals With Postprandial Glucose Level Decreases (PGD) Below Baseline
eFigure 4. Stratified Associations of Postprandial Glucose Level Decreases (PGD) at 2 to 3 Hours With Appetite Measures by Mealtime
eFigure 5. Stratified Associations of Postprandial Glucose Level Decreases (PGD) at 2 to 3 Hours With Appetite Measures by Glycemic Status
eFigure 6. Stratified Associations of Postprandial Glucose Level Decreases (PGD) at 2 to 3 Hours With Appetite Measures by Body Mass Index Groups

This supplemental material has been provided by the authors to give readers additional information about their work.

© 2026 Yao et al. *JAMA Network Open*.

eMethods

Participant exclusion criteria

Participant exclusion criteria included a known sensitivity to medical-grade adhesive, bleeding disorder, severe mental health condition, and a history of cardiovascular diseases, diabetes, cancer, kidney failure, or thyroid diseases.

Reminders for smartphone-based surveys

For the six times daily smartphone-based ecological momentary assessment (EMA) surveys, reminders spaced 10 minutes apart were sent via app push notifications before participants responded to the EMA. Up to four reminders were sent for each of the first five EMAs and up to two for the 6th EMA of the day.

Alternative premeal baseline glucose level for sensitivity analyses

For the main analysis, we computed premeal baseline glucose level specific to each meal, using the mean glucose level during 30 minutes before the meal. Besides this, we also conducted sensitivity analyses using time-window-specific premeal baseline glucose level (i.e., one premeal baseline glucose level for meals consumed during the breakfast time window, one for meals during the lunch time window, and one for meals during the dinner time window). The time-window-specific premeal baseline glucose level was computed by averaging all the mean 30-minute premeal glucose level across all meals consumed in the corresponding time window. The resulted associations between postprandial glucose level decreases and appetite measures, based on the time-window-specific premeal baseline glucose levels, can be found in Supplementary eTable 4.

Bootstrapping samples for computing confidence intervals for Spearman correlations

For each Spearman correlation coefficient between postprandial glucose measures and appetite measures (meals as observations), confidence interval was computed using 10,000 bootstrapping samples of the original meal data to assess statistical significance due to the clustered nature of the meal data. Steps to generate a bootstrapping sample included (1) resampling with replacement to create a random list of 895 participants from the original participant list and (2) extracting and aggregating the meal data of each participant in the randomly sampled list. Subsequently, the point estimate of each correlation coefficient was computed for each bootstrapping sample to approximate the correlation coefficient distribution to obtain the 95% confidence interval.

Measures of temporal lifestyle factors and postprandial satiety for sensitivity analyses

At the baseline visit, we fitted an accelerometer (AX3; Axivity Ltd) with the paired Axivity wristband on each participant's non-dominant wrist. Throughout the free-living days, the accelerometer automatically collected the participant's movements at a sampling frequency of 100 Hz. The accelerometer data were masked to participants. We used R package GGIR (version 3.1.5) to process raw accelerometer data, which involved auto-signal calibration and non-wear detection described elsewhere[1–3]. Subsequently, the accelerometer signals were aggregated into 5-second epoch data in 'Euclidean Norm Minus One' (ENMO). For each meal, the 5-second epoch data spanning 24 hours before and 2 hours after each meal (18,720 data points per meal) with movement labels were extracted to derive meal-specific movement measures by summing the duration for each movement type within each targeted timeframe. The movement labels/types included light intensity physical activity using ENMO (lower) threshold of 40 milligravity, moderate-to-vigorous intensity physical activity using ENMO (lower) threshold of 100 milligravity, and sleep using the HDCZA guider [2,4]. The derived measures for analyses included light intensity physical activity, moderate-to-vigorous intensity physical activity, and sleep during 24 hours before the meal, as well as light intensity physical activity and moderate-to-vigorous intensity physical activity within 2 hours after the meal.

The smartphone app-based EMA surveys delivered six times per day distributed across the day were used to collect the dietary information. Meal composition was collected using pre-defined Singapore local food group lists and check-all-that-apply questions (e.g., 'What foods were part of your meal?'). The meal composition collected for the sensitivity analysis included refined grains (e.g., white rice, noodles, pasta, bread, or cereal), whole grains (e.g., brown or wholegrain rice, noodles, pasta, bread, or cereal), seafood, chicken, red meat (e.g., beef, pork, mutton, lamb), eggs, dairy products (e.g., milk, yogurt, cheese), soy food (e.g., tofu and tempeh),

© 2026 Yao et al. *JAMA Network Open*.

beans, peas, nuts, seeds (e.g., peanuts, dahl, sambar), vegetables, fruit, deep fried foods (e.g., puffs, samosas, French fries, fried chicken), and sweet desserts, as well as various sugary and non-sugary beverages. Other food items that participants could not match directly to the pre-defined food group were recorded using free texts. These free-text food entries were independently examined by two researchers and were mapped to or merged with the closest food groups in the pre-defined lists, with disagreements resolved by consensus or by the decision of a third researcher. For each meal, we also collected participants' postmeal satiety for each meal using responses to 'How full did you feel after eating your meal?' with a 6-point Likert scale (1 = still hungry and 6 = extremely full).

References

1. van Hees VT, Gorzelniak L, Dean León EC, et al. Separating Movement and Gravity Components in an Acceleration Signal and Implications for the Assessment of Human Daily Physical Activity. *PLoS One*. 2013;8(4):e61691. doi:10.1371/journal.pone.0061691
2. Migueles JH, Rowlands A V., Huber F, Sabia S, van Hees VT. GGIR: A Research Community–Driven Open Source R Package for Generating Physical Activity and Sleep Outcomes From Multi-Day Raw Accelerometer Data. *J Meas Phys Behav*. 2019;2(3):188-196. doi:10.1123/jmpb.2018-0063
3. van Hees VT, Fang Z, Langford J, et al. Autocalibration of accelerometer data for free-living physical activity assessment using local gravity and temperature: an evaluation on four continents. *J Appl Physiol (1985)*. 2014;117(7):738-744. doi:10.1152/japplphysiol.00421.2014
4. van Hees VT, Sabia S, Jones SE, et al. Estimating sleep parameters using an accelerometer without sleep diary. *Sci Rep*. 2018;8(1):12975. doi:10.1038/s41598-018-31266-z

© 2026 Yao et al. *JAMA Network Open*.

SUPPLEMENTARY TABLES

eTable 1. Meal characteristics, overall, and by mealtime.

Characteristics	Total	Mealtime		
		05:00-11:00 h	11:00-17:00 h	17:00-24:00 h
Number meals	7650 (100%)	2163 (28%)	2850 (37%)	2637 (34%)
Meal glucose measures				
Premeal baseline glucose level, mmol/l	5.3 (4.8, 6.0)	5.2 (4.8, 5.8)	5.3 (4.8, 6.0)	5.5 (4.9, 6.2)
Postprandial glucose iAUC at 0-2 h, mmol/l*h	1.9 (0.6, 3.5)	1.7 (0.7, 2.9)	2.2 (0.6, 4.0)	1.8 (0.4, 3.6)
Postprandial glucose peak at 0-2 h, mmol/l	7.4 (6.5, 8.5)	7.2 (6.3, 8.2)	7.6 (6.6, 8.7)	7.5 (6.6, 8.6)
Postprandial glucose rise magnitude, %	36.3 (16.5, 60.3)	36.4 (20.2, 56.0)	39.4 (16.7, 65.0)	33.3 (13.3, 58.3)
Postprandial glucose nadir at 2-3 h, mmol/l	5.2 (4.7, 5.8)	5.0 (4.5, 5.5)	5.3 (4.8, 5.9)	5.4 (4.9, 6.0)
PGD magnitude, %	2.0 (-10.0, 13.5)	4.3 (-4.9, 13.2)	0.0 (-12.7, 12.8)	1.8 (-11.6, 14.3)
PGD below baseline: yes	4121 (54%)	1324 (61%)	1404 (49%)	1393 (53%)
Appetite measures				
Time until next meal, minutes	405 (300, 690)	301 (246, 445)	385 (325, 435)	795 (714, 860)
Time until next meal or snack, minutes	370 (273, 650)	295 (240, 390)	360 (289, 420)	778 (680, 848)
Postprandial satiety level	4 (3, 4)	4 (3, 4)	4 (3, 5)	4 (4, 5)
Premeal hunger level	2 (1, 3)	2 (1, 3)	2 (1, 3)	2 (1, 3)
Postprandial hunger level at 2-3 h	1 (0, 2)	1 (0, 2)	1 (0, 2)	1 (0, 1)
Postprandial hunger increase at 2-3 h	-1 (-2, 0)	-1 (-2, 0)	-1 (-2, 0)	-1 (-2, 0)
Postprandial hunger level at 3-4 h	1 (0, 3)	2 (1, 3)	1 (0, 2)	1 (0, 2)
Postprandial hunger increase at 3-4 h	0 (-2, 0)	0 (-1, 0)	0 (-1, 0)	0 (-2, 0)
Availability of appetite measures				
N meals with time until next meal	5456	1932	2017	1507
N meals with time until next meal or snack	5456	1932	2017	1507
N meals with postprandial satiety level	7637	2158	2846	2633
N meals with premeal hunger level	4369	682	1949	1738
N meals with postprandial hunger level 2-3h	2636	829	1029	778
N meal with postprandial hunger increase at 2-3h	1265	210	643	412
N meal with postprandial hunger level at 3-4h	2419	850	1183	386
N meal with postprandial hunger increase at 3-4h	1354	231	855	268

Data in N, N (%), or median (IQR). PGD: postprandial glucose level decreases.

© 2026 Yao et al. *JAMA Network Open*.

eTable 2. Postprandial glucose level decreases stratified by participant glycemic status, weight status, and mealtime.

	Meals: PGD magnitude (% baseline)			Meals: N (%) with PGD below baseline	Participants: within-person proportion of meals with PGD below baseline (%)		
	25th percentile	50th percentile	75th percentile		25th percentile	50th percentile	75th percentile
All participants	-10.0	2.0	13.5	4121 (54%)	36.4	50.0	73.3
Glycemic status							
Normoglycemia	-9.4	2.1	13.4	3034 (54%)	37.5	53.8	75.0
Pre-diabetes	-12.5	1.6	13.8	1087 (52%)	33.3	50.0	66.7
BMI group, kg/m^2							
< 23	-9.8	1.8	12.9	1920 (53%)	35.7	50.0	68.8
>= 23 and < 27.5	-9.5	2.0	13.7	1330 (55%)	37.5	53.8	75.0
>= 27.5	-11.4	2.7	14.3	871 (55%)	38.5	50.0	77.8
Mealtime							
05:00-11:00 h	-4.9	4.3	13.2	1324 (61%)	33.3	66.7	100.0
11:00-17:00 h	-12.7	0.0	12.8	1404 (49%)	25.0	50.0	80.0
17:00-24:00 h	-11.6	1.8	14.3	1393 (53%)	25.0	50.0	100.0

PGD: postprandial glucose level decreases.

© 2026 Yao et al. *JAMA Network Open*.

eTable 3. Meal characteristics: appetite measures by postprandial glucose level decrease below baseline.

Appetite measures	PGD below baseline	
	No	Yes
Median (IQR):		
Time until next meal, minutes	425 (325, 714)	380 (285, 660)
Time until next meal or snack, minutes	395 (296, 694)	347 (263, 604)
Postprandial hunger level 2-3 h	1 (0, 2)	1 (0, 2)
Postprandial hunger increase 2-3 h	-1 (-2, 0)	-1 (-2, 0)
Postprandial hunger level 3-4 h	1 (0, 2)	1 (1, 3)
Postprandial hunger increase 3-4 h	0 (-2, 0)	0 (-1, 0)
Mean (SD):		
Time until next meal, minutes	501.8 (222.8)	463.0 (225.5)
Time until next meal or snack, minutes	472.9 (226.4)	435.0 (224.2)
Postprandial hunger level 2-3 h	1.1 (1.1)	1.3 (1.2)
Postprandial hunger increase 2-3 h	-1.3 (1.5)	-1.0 (1.6)
Postprandial hunger level 3-4 h	1.4 (1.3)	1.7 (1.4)
Postprandial hunger increase 3-4 h	-0.8 (1.6)	-0.6 (1.5)

PGD: postprandial glucose level decreases.

© 2026 Yao et al. *JAMA Network Open*.

eTable 4. Associations of postprandial glucose level decreases with appetite measures, main and sensitivity analyses.

Postprandial appetite outcomes	PGD magnitude (%10 baseline)		PGD below baseline	
	β (95% CI)	P value	β (95% CI)	P value
Overall association: main model (source data for Figure 3)				
Hunger level at 2-3 h	0.05 (0.03, 0.07)	<.001	0.22 (0.12, 0.31)	<.001
Hunger increase at 2-3 h	0.08 (0.04, 0.12)	<.001	0.30 (0.13, 0.47)	<.001
Hunger level at 3-4 h	0.09 (0.06, 0.13)	<.001	0.30 (0.17, 0.44)	<.001
Hunger increase at 3-4 h	0.07 (0.02, 0.12)	.005	0.25 (0.05, 0.45)	.01
Time to next meal, min	-6.30 (-8.88, -3.72)	<.001	-27.30 (-36.90, -17.71)	<.001
Time to next meal or snack, min	-6.54 (-9.13, -3.94)	<.001	-26.82 (-36.79, -16.84)	<.001
Within-person association (source data for Figure 3)				
Hunger level at 2-3 h	0.04 (0.02, 0.06)	<.001	0.16 (0.08, 0.25)	<.001
Hunger increase at 2-3 h	0.08 (0.01, 0.14)	.02	0.36 (0.14, 0.57)	.001
Hunger level at 3-4 h	0.08 (0.04, 0.11)	<.001	0.20 (0.08, 0.33)	.002
Hunger increase at 3-4 h	0.10 (0.02, 0.18)	.02	0.22 (-0.12, 0.55)	.21
Time to next meal, min	-6.04 (-8.99, -3.09)	<.001	-25.53 (-36.14, -14.91)	<.001
Time to next meal or snack, min	-6.57 (-9.56, -3.59)	<.001	-25.00 (-36.02, -13.98)	<.001
Overall association: main model + adjusting for postprandial glucose iAUC at 0-2 h				
Hunger level at 2-3 h	0.04 (0.02, 0.07)	.002	0.18 (0.07, 0.28)	.001
Hunger increase at 2-3 h	0.03 (-0.02, 0.08)	.23	0.14 (-0.05, 0.33)	.144
Hunger level at 3-4 h	0.10 (0.05, 0.14)	<.001	0.27 (0.12, 0.42)	.001
Hunger increase at 3-4 h	0.04 (-0.02, 0.10)	.23	0.14 (-0.09, 0.37)	.24
Time to next meal, min	-7.03 (-10.08, -3.98)	<.001	-27.78 (-37.89, -17.67)	<.001
Time to next meal or snack, min	-7.56 (-10.63, -4.50)	<.001	-27.68 (-38.18, -17.19)	<.001
Overall association: main model + adjusting for postprandial satiety				
Hunger level at 2-3 h	0.04 (0.02, 0.06)	<.001	0.17 (0.08, 0.26)	<.001
Hunger increase at 2-3 h	0.06 (0.02, 0.11)	.002	0.25 (0.08, 0.42)	.004
Hunger level at 3-4 h	0.08 (0.05, 0.12)	<.001	0.27 (0.14, 0.41)	<.001
Hunger increase at 3-4 h	0.06 (0.01, 0.11)	.01	0.23 (0.03, 0.43)	.03
Time to next meal, min	-5.74 (-8.26, -3.22)	<.001	-24.95 (-34.37, -15.54)	<.001
Time to next meal or snack, min	-5.98 (-8.53, -3.42)	<.001	-24.46 (-34.32, -14.60)	<.001
Overall association: main model + adjusting for physical activity and sleep †				
Hunger level at 2-3 h	0.05 (0.03, 0.07)	<.001	0.22 (0.13, 0.31)	<.001
Hunger increase at 2-3 h	0.08 (0.04, 0.12)	<.001	0.32 (0.15, 0.49)	<.001
Hunger level at 3-4 h	0.09 (0.06, 0.13)	<.001	0.30 (0.16, 0.43)	<.001
Hunger increase at 3-4 h	0.07 (0.02, 0.12)	.005	0.26 (0.05, 0.46)	0.01
Time to next meal, min	-6.40 (-8.99, -3.81)	<.001	-27.61 (-37.25, -17.97)	<.001

© 2026 Yao et al. *JAMA Network Open*.

Time to next meal or snack, min	-6.67 (-9.27, -4.06)	<.001	-27.16 (-37.17, -17.16)	<.001
Overall association: main model + adjusting for physical activity, sleep, and meal composition †				
Hunger level at 2-3 h	0.04 (0.02, 0.07)	<.001	0.20 (0.10, 0.29)	<.001
Hunger increase at 2-3 h	0.07 (0.03, 0.12)	.001	0.30 (0.13, 0.47)	<.001
Hunger level at 3-4 h	0.09 (0.05, 0.12)	<.001	0.30 (0.17, 0.44)	<.001
Hunger increase at 3-4 h	0.07 (0.02, 0.12)	.007	0.28 (0.07, 0.48)	.008
Time to next meal, min	-5.67 (-8.14, -3.19)	<.001	-23.98 (-33.17, -14.80)	<.001
Time to next meal or snack, min	-6.05 (-8.54, -3.56)	<.001	-23.95 (-33.45, -14.46)	<.001
Overall association: sensitivity model based on the three time-window-specific baseline glucose (average 30-min premeal glucose across all meals for the time window)				
Hunger level at 2-3 h	0.05 (0.03, 0.07)	<.001	0.21 (0.12, 0.30)	<.001
Hunger increase at 2-3 h	0.07 (0.03, 0.12)	.001	0.29 (0.13, 0.46)	.001
Hunger level at 3-4 h	0.09 (0.06, 0.13)	<.001	0.30 (0.16, 0.44)	<.001
Hunger increase at 3-4 h	0.06 (0.02, 0.11)	.007	0.21 (0.01, 0.41)	.04
Time to next meal, min	-6.56 (-9.22, -3.90)	<.001	-26.77 (-36.46, -17.07)	<.001
Time to next meal or snack, min	-6.93 (-9.61, -4.25)	<.001	-28.40 (-38.64, -18.17)	<.001
Overall association: main model but restricted to n = 1981 meals with premeal hunger measure within 1 h (instead of 2 h) before mealtime				
Hunger level at 2-3 h	0.06 (0.03, 0.10)	0.001	0.32 (0.15, 0.49)	<.001
Hunger increase at 2-3 h	0.06 (-0.00, 0.12)	.06	0.23 (-0.01, 0.48)	.07
Hunger level at 3-4 h	0.13 (0.06, 0.21)	<.001	0.47 (0.16, 0.78)	.003
Hunger increase at 3-4 h	0.22 (0.13, 0.30)	<.001	0.74 (0.39, 1.09)	<.001
Time to next meal, min	-5.67 (-10.80, -0.53)	.03	-28.14 (-44.28, -12.00)	.001
Time to next meal or snack, min	-6.78 (-11.85, -1.70)	.009	-30.15 (-48.30, -11.99)	.001

† The adjusted sleep measure was sleep duration during the 24 hours before the meal; The adjusted physical activity measures were light intensity physical activity and moderate-to-vigorous intensity physical activity during 24 hours before the meal and these physical activity during the 2-hour postprandial period; The adjusted meal composition included refined grains, whole grains, seafood, chicken, red meat, eggs, dairy, beans or nuts, vegetables, fruit, deep fried foods, sweet desserts, sugary drinks, and non-sugary drinks.
PGD: postprandial glucose level decreases.

© 2026 Yao et al. *JAMA Network Open*.

eTable 5. Associations of postprandial glucose level decreases with time to the next meal: main model vs sensitivity models with log-transformation and models with Gamma distribution.

Model specification	Percent change (multiplicative) in time to the next meal (95% CI)	Absolute change (additive in minutes) of time to the next meal at mean (452 minutes); 95% CI	Absolute change (additive in minutes) of time to the next meal at median (405 minutes); 95% CI
Exposure: PGD magnitude (%10 baseline)			
Main analysis: no transformation before applying GEE with Gaussian identity link	NA	-6.30 (-8.88, -3.72)	
Nature log transformation before applying GEE with Gaussian identity link	-0.015 (-0.020, -0.009)	-7.08 (-9.65, -4.50)	-5.98 (-8.14, -3.80)
No transformation before applying GEE with Gamma log link	-0.012 (-0.017, -0.007)	-5.90 (-8.30, -3.48)	-4.98 (-7.00, -2.94)
Exposure: PGD below baseline: yes			
Main analysis: no transformation before applying GEE with Gaussian identity link	NA	-27.30 (-36.90, -17.71)	
Nature log transformation before applying GEE with Gaussian identity link	-0.066 (-0.086, -0.047)	-31.88 (-41.11, -22.45)	-26.90 (-34.68, -18.95)
No transformation before applying GEE with Gamma log link	-0.058 (-0.078, -0.038)	-28.05 (-37.46, -18.45)	-23.67 (-31.60, -15.56)

PGD: postprandial glucose level decreases.

© 2026 Yao et al. *JAMA Network Open*.

eTable 6. Associations of postprandial glucose level decreases with time to the next meal or snacks: main model vs sensitivity models with log-transformation and models with Gamma distribution.

**Model specification**	**Percent change (multiplicative) in time to the next meal or snack (95% CI)**	**Absolute change (additive in minutes) of time to the next meal or snack at mean (452 minutes); 95% CI**	**Absolute change (additive in minutes) of time to the next meal or snack at median (370 minutes); 95% CI**
Exposure: PGD magnitude (%10 baseline)			
Main analysis: no transformation before applying GEE with Gaussian identity link	NA	-6.54 (-9.13, -3.94)	
Nature log transformation before applying GEE with Gaussian identity link	-0.016 (-0.021, -0.010)	-7.10 (-9.63, -4.56)	-5.81 (-7.88, -3.73)
No transformation before applying GEE with Gamma log link	-0.013 (-0.018, -0.008)	-6.02 (-8.36, -3.67)	-4.93 (-6.84, -3.01)
Exposure: PGD below baseline: yes			
Main analysis: no transformation before applying GEE with Gaussian identity link	NA	-26.82 (-36.79, -16.84)	
Nature log transformation before applying GEE with Gaussian identity link	-0.066 (-0.086, -0.045)	-29.72 (-39.00, -20.24)	-24.33 (-31.92, -16.57)
No transformation before applying GEE with Gamma log link	-0.059 (-0.080, -0.038)	-26.79 (-36.09, -17.29)	-21.93 (-29.54, -14.15)

PGD: postprandial glucose level decreases.

© 2026 Yao et al. *JAMA Network Open*.

eTable 7. Interactions by mealtime, glycemic status, and weight status for associations between postprandial glucose level decreases and appetite.

Postprandial appetite outcomes	Interaction terms with PGD †	PGD magnitude (%10 baseline)		PGD below baseline	
		β (95% CI)	P value (p-interaction)	β (95% CI)	P value (p-interaction)
Hunger level at 2-3 h	Mealtime: 11:00-17:00 h	-0.01 (-0.07, 0.05)	.71	0.10 (-0.12, 0.32)	.35
	Mealtime: 17:00-24:00 h	-0.02 (-0.08, 0.05)	.60	-0.04 (-0.27, 0.20)	.77
	Glycemic status: pre-diabetes	-0.01 (-0.05, 0.04)	.68	0.03 (-0.16, 0.23)	.74
	BMI: ≥23 & <27.5, and ≥27.5	0.03 (-0.02, 0.08)	.28	0.01 (-0.21, 0.23)	.94
	BMI: ≥27.5	-0.03 (-0.08, 0.02)	.29	-0.13 (-0.37, 0.11)	.28
Hunger increase at 2-3 h	Mealtime: 11:00-17:00 h	0.14 (0.01, 0.27)	.04	0.22 (-0.26, 0.71)	.37
	Mealtime: 17:00-24:00 h	0.19 (0.06, 0.32)	.003	0.25 (-0.25, 0.75)	.33
	Glycemic status: pre-diabetes	0.03 (-0.07, 0.12)	.58	0.06 (-0.33, 0.46)	.76
	BMI: ≥23 & <27.5, and ≥27.5	-0.02 (-0.12, 0.08)	.68	-0.14 (-0.51, 0.24)	.48
	BMI: ≥27.5	-0.07 (-0.18, 0.04)	.23	-0.30 (-0.78, 0.18)	.21
Hunger level at 3-4 h	Mealtime: 11:00-17:00 h	-0.03 (-0.11, 0.06)	.51	-0.03 (-0.34, 0.28)	.83
	Mealtime: 17:00-24:00 h	-0.04 (-0.15, 0.06)	.40	-0.14 (-0.54, 0.26)	.49
	Glycemic status: pre-diabetes	0.00 (-0.07, 0.08)	.92	-0.16 (-0.45, 0.12)	.27
	BMI: ≥23 & <27.5, and ≥27.5	0.00 (-0.08, 0.09)	.99	-0.09 (-0.41, 0.24)	.59
	BMI: ≥27.5	-0.06 (-0.15, 0.04)	.24	-0.25 (-0.61, 0.12)	.18
Hunger increase at 3-4 h	Mealtime: 11:00-17:00 h	-0.02 (-0.17, 0.12)	.73	0.15 (-0.42, 0.72)	.60
	Mealtime: 17:00-24:00 h	0.11 (-0.06, 0.28)	.22	0.30 (-0.41, 1.00)	.41
	Glycemic status: pre-diabetes	0.08 (-0.02, 0.18)	.12	0.09 (-0.34, 0.51)	.70
	BMI: ≥23 & <27.5, and ≥27.5	-0.09 (-0.20, 0.02)	.12	-0.45 (-0.92, 0.02)	.06
	BMI: ≥27.5	-0.07 (-0.21, 0.06)	.30	-0.44 (-1.02, 0.15)	.15

(Continue in the next page)

© 2026 Yao et al. *JAMA Network Open*.

eTable 7 (Continued)

Postprandial appetite outcomes	Interaction terms with PGD †	PGD magnitude (%10 baseline)		PGD below baseline	
		β (95% CI)	P value (p-interaction)	β (95% CI)	P value (p-interaction)
Time to next meal, min	Mealtime: 11:00-17:00 h	2.21 (-3.19, 7.61)	.42	15.88 (-3.97, 35.73)	.12
	Mealtime: 17:00-24:00 h	-3.19 (-9.57, 3.20)	.33	2.74 (-21.41, 26.89)	.82
	Glycemic status: pre-diabetes	2.58 (-2.64, 7.80)	.33	2.19 (-19.59, 23.98)	.84
	BMI: ≥23 & <27.5, and ≥27.5	-2.59 (-8.35, 3.16)	.38	7.06 (-14.74, 28.86)	.53
	BMI: ≥27.5	-0.26 (-6.30, 5.78)	.93	8.51 (-14.36, 31.38)	.47
Time to next meal or snack, min	Mealtime: 11:00-17:00 h	-0.07 (-5.21, 5.07)	.98	3.94 (-15.07, 22.94)	.68
	Mealtime: 17:00-24:00 h	-5.04 (-11.84, 1.75)	.15	-7.71 (-34.14, 18.72)	.57
	Glycemic status: pre-diabetes	1.96 (-3.27, 7.19)	.46	2.85 (-19.84, 25.55)	.81
	BMI: ≥23 & <27.5, and ≥27.5	-0.51 (-6.64, 5.61)	.87	4.65 (-17.69, 26.98)	.68
	BMI: ≥27.5	-1.62 (-7.30, 4.06)	.58	3.36 (-19.96, 26.68)	.78

† Reference group for mealtime: 05:00-11:00 h; glycemic status: normoglycemia; BMI: <23 kg/m^2

PGD: postprandial glucose level decreases.

© 2026 Yao et al. *JAMA Network Open*.

SUPPLEMENTARY FIGURES

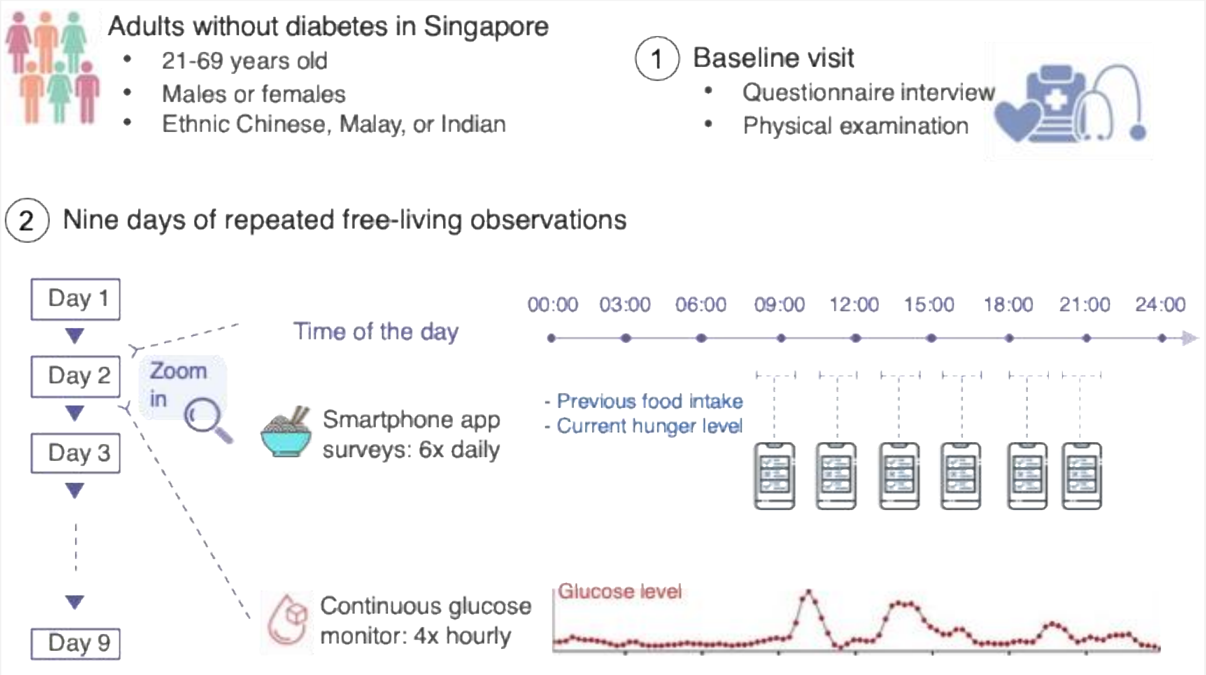

eFigure 1. Study design.

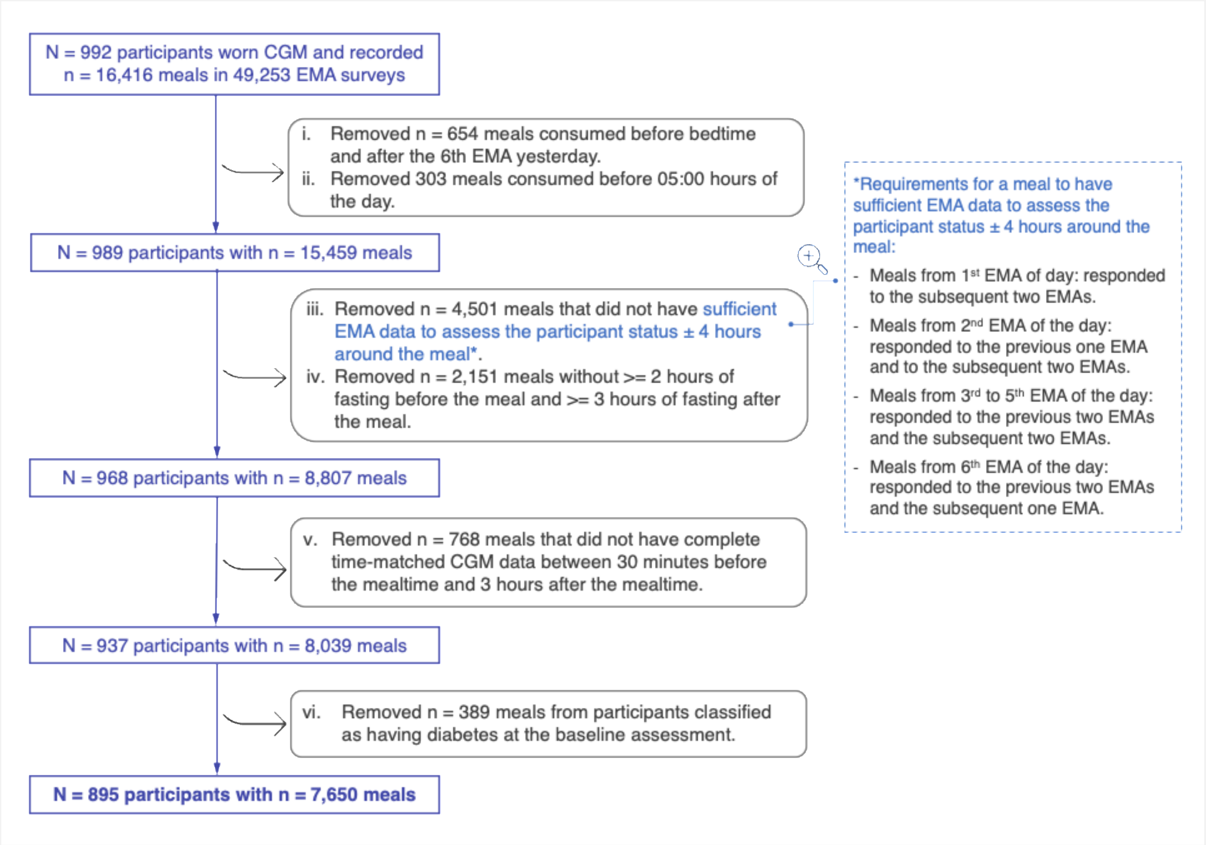

eFigure 2. Meal and participant inclusion and flowchart.

CGM: continuous glucose monitoring; EMA: ecological momentary assessment surveys via smartphone.

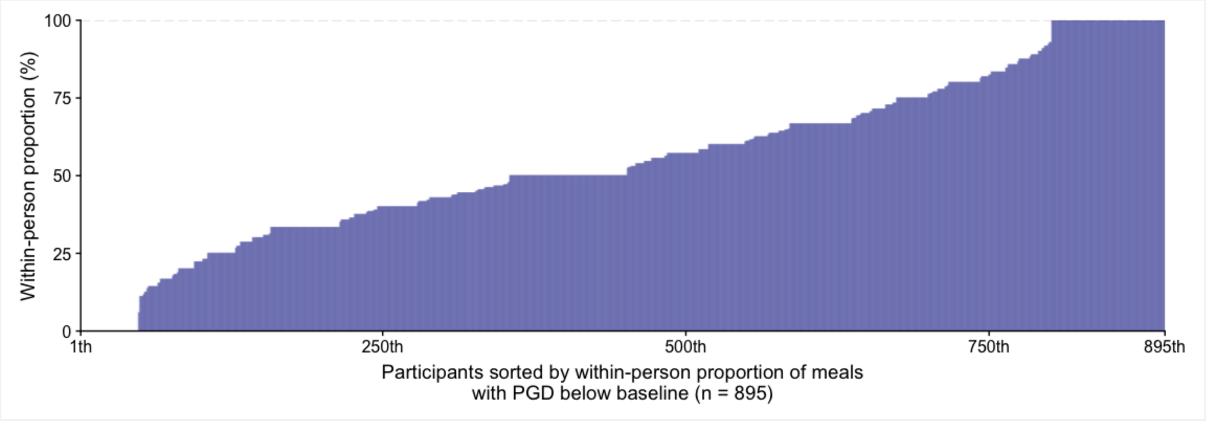

eFigure 3. Bar plot of participants' proportion of meals with postprandial glucose level decreases (PGD) below baseline.

The proportion of meals with PGD below baseline among the 895 participants ranged from 0 to 100%. Each column represents one participant, sorted from left to right by the increasing within-person proportion of meals with PGD below baseline. There were 48 participants with 0% of meals with PGD below baseline, and 94 participants with 100% of meals with PGD below baseline.

© 2026 Yao et al. *JAMA Network Open*.

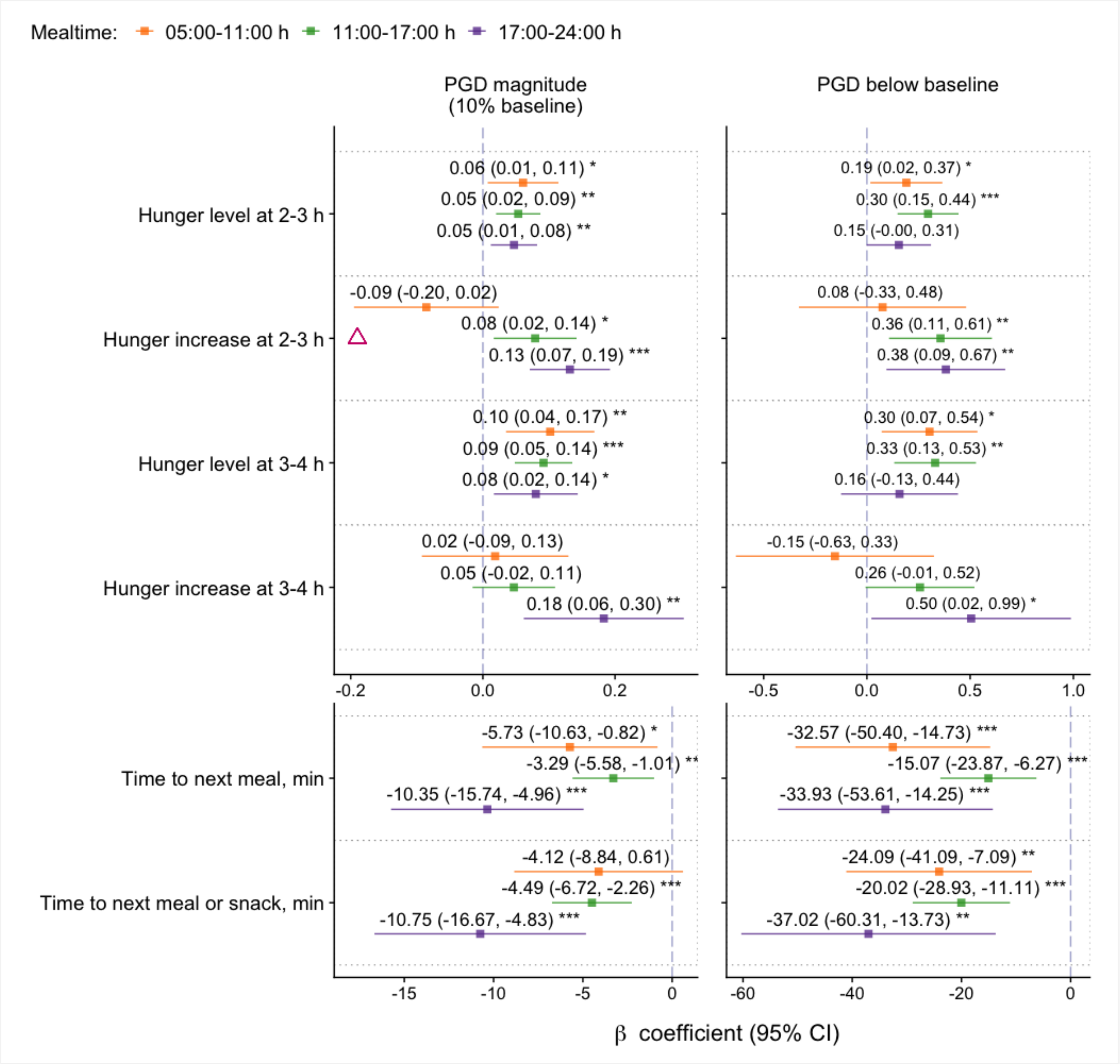

eFigure 4. Stratified associations of postprandial glucose level decreases (PGD) at 2 to 3 hours with appetite measures by mealtime.

The associations were estimated using generalized estimating equation models, adjusted for age, sex, ethnicity, glycemic status, BMI, cigarette smoking, alcohol consumption, and education level. The symbol △ marks a significant PGD magnitude x mealtime interaction for the hunger increase at 2-3 h. P-value: <.05, <.01**, <.001***.*

© 2026 Yao et al. *JAMA Network Open*.

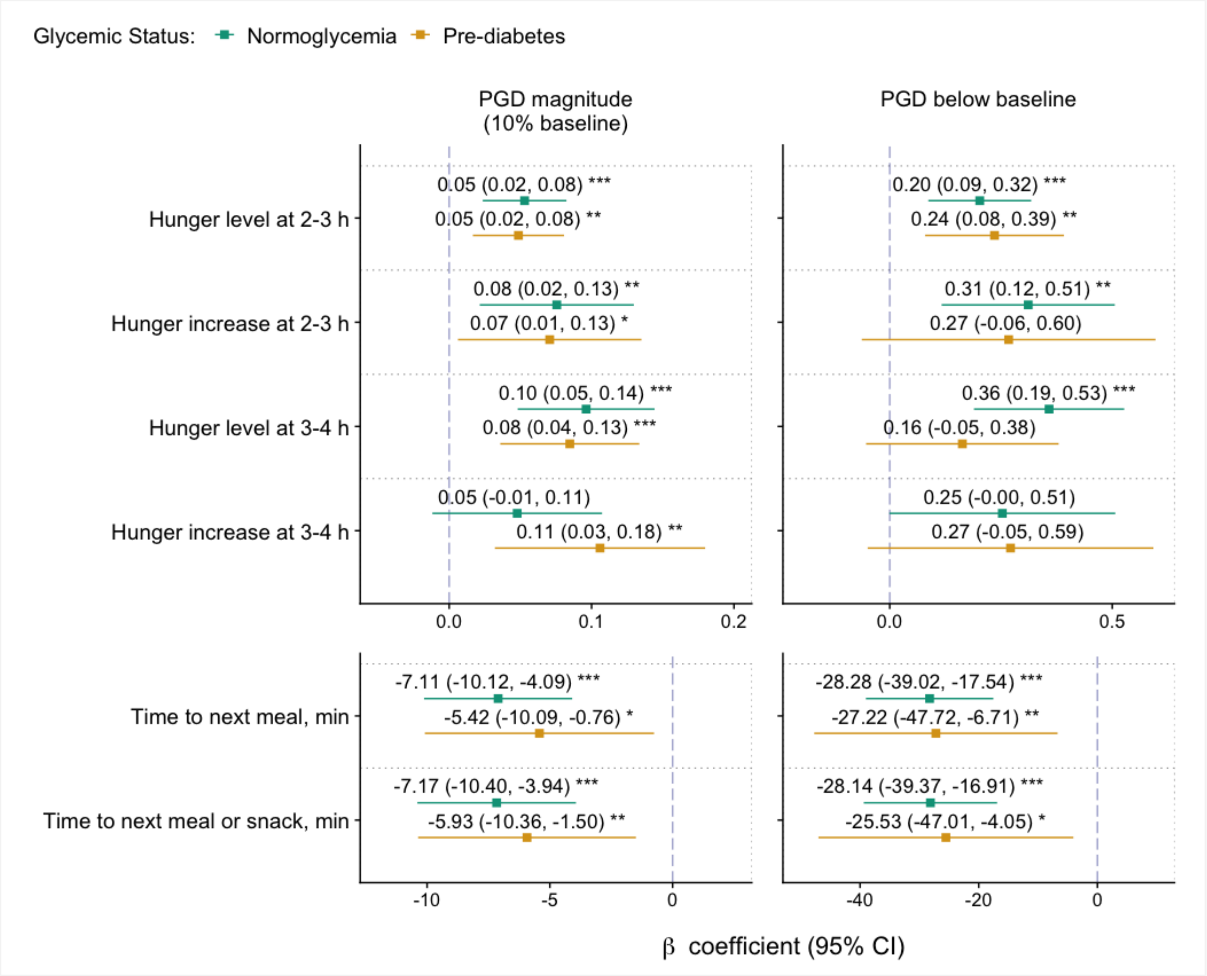

eFigure 5. Stratified associations of postprandial glucose level decreases (PGD) at 2 to 3 hours with appetite measures by glycemic status.

The associations were estimated using generalized estimating equation models, adjusted for age, sex, ethnicity, BMI, cigarette smoking, alcohol consumption, education level, and mealtime. P-value: <.05, <.01**, <.001***.*

© 2026 Yao et al. *JAMA Network Open*.

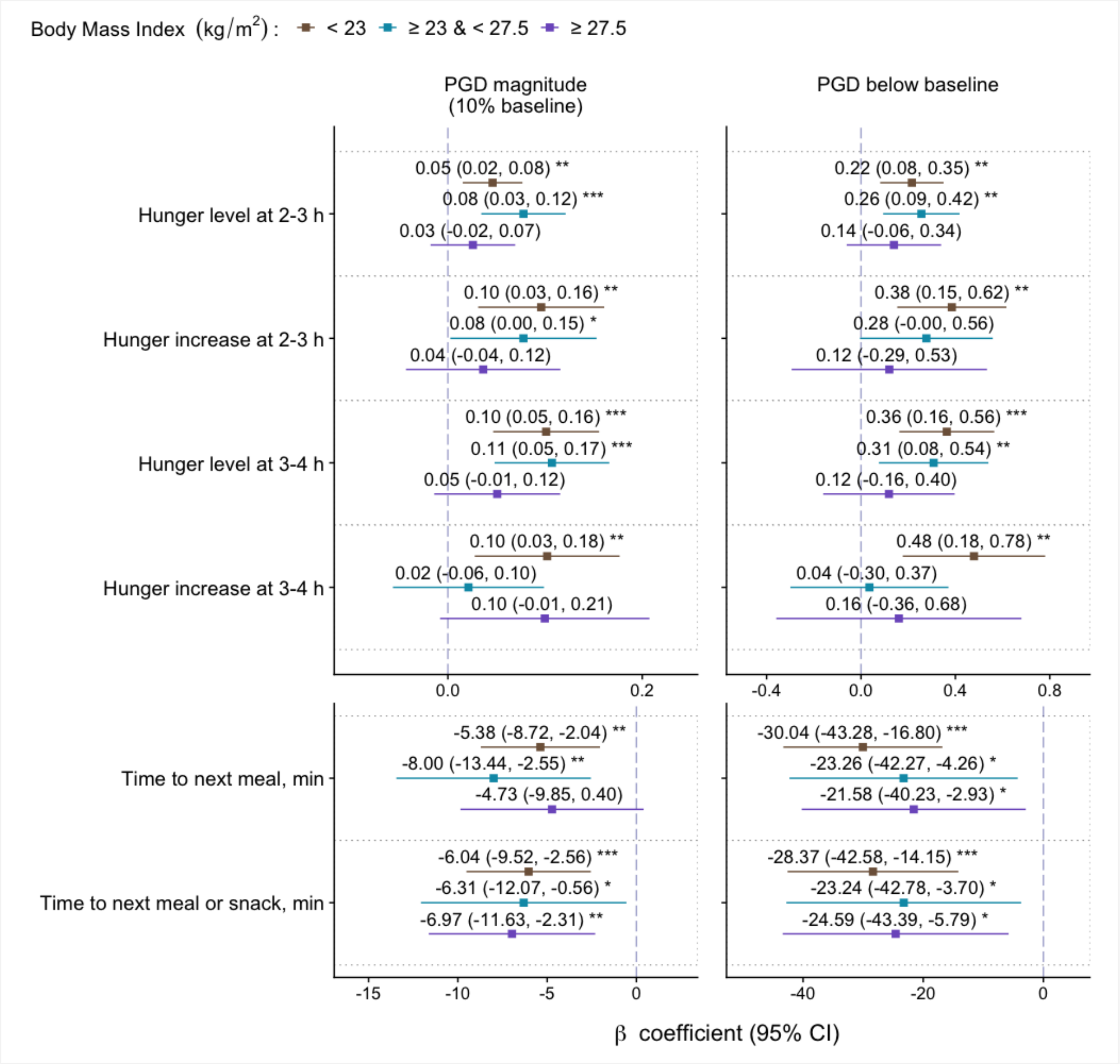

eFigure 6. Stratified associations of postprandial glucose level decreases (PGD) at 2 to 3 hours with appetite measures by body mass index groups.

The associations were estimated using generalized estimating equation models, adjusted for age, sex, ethnicity, glycemic status, cigarette smoking, alcohol consumption, education level, and mealtime. P-value: <.05, <.01**, <.001***.*

© 2026 Yao et al. *JAMA Network Open*.
