# Supplementary material for: Postprandial Glucose Level Decreases and Appetite in Adults Without Diabetes
Source: JAMA Netw Open. 2026 Mar 26;9(3):e263426. doi: 10.1001/jamanetworkopen.2026.3426 (PMC13022734; doi:10.1001/jamanetworkopen.2026.3426)
Supplement: Supplement 2. — Data Sharing Statement [file jamanetwopen-e263426-s002.pdf]

Data Sharing Statement

Yao. Postprandial Glucose Level Decreases and Appetite in Adults Without Diabetes. *JAMA Netw Open*. Published March 26, 2026. doi:10.1001/jamanetworkopen.2026.3426

Data
Data available: No

Additional Information
Explanation for why data not available: The data used for this study are held by the Saw Swee Hock School of Public Health at the National University of Singapore. The data can be released to bona fide researchers upon reasonable requests and agreements via https://blog.nus.edu.sg/sphs/data-and-samples-request/. The data release must conform to the Personal Data Protection Act in Singapore. The scripts for statistical analysis will be provided upon request to the corresponding author.
